# Multi-scale convolutional transformer network for motor imagery brain-computer interface

**DOI:** 10.1038/s41598-025-96611-5

**Published:** 2025-04-15

**Authors:** Wei Zhao, Baocan Zhang, Haifeng Zhou, Dezhi Wei, Chenxi Huang, Quan Lan

**Affiliations:** 1 Chengyi College, Jimei University, Xiamen, 361021 China; 2 School of Marine Engineering, Jimei University, Xiamen, 361021 China; 3 School of Informatics, Xiamen University, Xiamen, 361005 China; 4 Department of Neurology, Department of Neuroscience, School of Medicine, The First Affiliated Hospital of Xiamen University, Xiamen University, Xiamen, 361005 China; 5 Fujian Key Laboratory of Brain Tumors Diagnosis and Precision Treatment, Xiamen, 361005 China

**Keywords:** Brain-computer interface (BCI), Convolutional neural networks (CNNs), Electroencephalography (EEG), Motor imagery (MI), Transformer, Network models, Neuroscience, Brain-machine interface

## Abstract

Brain-computer interface (BCI) systems allow users to communicate with external devices by translating neural signals into real-time commands. Convolutional neural networks (CNNs) have been effectively utilized for decoding motor imagery electroencephalography (MI-EEG) signals in BCIs. However, traditional CNN-based methods face challenges such as individual variability in EEG signals and the limited receptive fields of CNNs. This study presents the Multi-Scale Convolutional Transformer (MSCFormer) model that integrates multiple CNN branches for multi-scale feature extraction and a Transformer module to capture global dependencies, followed by a fully connected layer for classification. The multi-branch multi-scale CNN structure effectively addresses individual variability in EEG signals, enhancing the model’s generalization capabilities, while the Transformer encoder strengthens global feature integration and improves decoding performance. Extensive experiments on the BCI IV-2a and IV-2b datasets show that MSCFormer achieves average accuracies of 82.95% (BCI IV-2a) and 88.00% (BCI IV-2b), with kappa values of 0.7726 and 0.7599 in five-fold cross-validation, surpassing several state-of-the-art methods. These results highlight MSCFormer’s robustness and accuracy, underscoring its potential in EEG-based BCI applications. The code has been released in https://github.com/snailpt/MSCFormer.

## Introduction

Brain-computer interface (BCI) technology has opened new avenues for direct communication between the brain and external devices^1^. BCI systems predominantly rely on various neural signal technologies, such as functional magnetic resonance imaging, electroencephalography (EEG), electrocorticography, and magnetoencephalography, to monitor and interpret brain activity patterns^2^. EEG technology, which records electrical activity in the brain via electrodes placed on the scalp, is particularly valued for its high temporal resolution, non-invasiveness, cost-effectiveness, and ease of use^3^. The motor imagery (MI) BCI paradigm is particularly noteworthy as it enables control of external devices through the mental simulation of specific movements (e.g., hand or foot movements) without actual execution. The MI-EEG paradigm has become a key technology in neurorehabilitation^4^, prosthetic control^5^, and human-computer interaction^6^.

Despite its promising applications, the MI-EEG paradigm faces significant challenges in accurately decoding user intentions^7^. EEG signals have a very low signal-to-noise ratio and are highly susceptible to interference, including electromyographic noise, environmental electromagnetic interference, and ocular artifacts, all of which degrade signal quality and decoding accuracy^8^. Additionally, when imagining the same motor task, EEG signals exhibit significant variability not only between individuals but also within the same individual at different times.

Traditional machine learning techniques have been widely applied to MI-EEG classification, typically employing a two-stage pipeline of feature extraction and classifier training. Among these, common spatial pattern (CSP) and its extension, filter bank CSP (FBCSP), are widely used for spatial filtering and frequency-specific feature extraction^9,10^. Other feature extraction methods, such as power spectral density, wavelet transform, and short-time Fourier transform (STFT), have been explored to characterize EEG signals in different domains^11–13^, while non-linear measures like approximate entropy and fractal dimension aim to capture signal complexity^14,15^. Classification is then performed using machine learning algorithms, including support vector machines, linear discriminant analysis, and k-nearest neighbors^16–18^.

Despite their effectiveness, traditional approaches heavily rely on handcrafted features, making them sensitive to inter-subject variability and limiting their adaptability. Furthermore, the separation of feature extraction and classification hinders joint optimization, potentially reducing classification performance^19,20^. Deep learning (DL)-based approaches overcome these limitations by automatically learning task-relevant features from raw EEG signals, enabling end-to-end optimization and reducing reliance on manual feature engineering^21–23^.

CNNs have become the dominant architecture in DL-based MI-EEG decoding, with recent studies investigating various convolution techniques, kernel sizes, and network depths. For instance, Schirrmeister et al.^20^ developed end-to-end ConvNets that outperformed FBCSP-based methods by directly learning hierarchical feature representations from raw EEG signals. Lawhern et al.^24^ introduced EEGNet, a compact CNN architecture with strong generalization across multiple BCI paradigms. To enhance feature extraction, Mane et al.^25^ proposed a filter-bank CNN that applies bandpass filtering (BPF) to EEG signals, while Wang et al.^26^ and Lee & Choi^27^ explored transform-domain CNNs, leveraging short-time Fourier transform (STFT) and continuous wavelet transform for MI-EEG classification.

Recent advancements show that while single-scale CNNs (SSCNNs) perform well in MI-EEG decoding, their limited ability to capture the complex spatiotemporal characteristics of EEG signals makes them less effective in handling inter-individual variability. Additionally, their restricted receptive field may limit the capture of long-range dependencies, potentially affecting decoding accuracy. LSTM-based models^28^ address this limitation by capturing temporal dependencies, but their sequential nature prevents efficient parallelization and can suffer from vanishing gradients in long EEG sequences. Meanwhile, Transformer-based models^29,30^ effectively capture global dependencies, but they may struggle to extract fine-grained local features, which are crucial for MI-EEG classification. Furthermore, training DL models require large amounts of labeled data, yet obtaining high-quality MI-EEG data is both time-consuming and resource-intensive due to the lengthy experimental protocols and high demands on subjects.

To tackle these challenges, this study proposes the Multi-Scale Convolutional Transformer (MSCFormer), which leverages CNNs for local spatial-temporal feature extraction and a Transformer encoder for global dependency modeling. The multi-scale CNN module addresses inter-subject variability by extracting features at different temporal scales, while the Transformer module mitigates CNN’s receptive field limitations by modeling long-range dependencies. Furthermore, data augmentation techniques are incorporated to enhance model generalization given the limited availability of EEG training data. Extensive experiments on the BCI IV-2a and IV-2b datasets validate the effectiveness of MSCFormer, demonstrating superior classification accuracy and robustness compared to existing methods. The following are the main contributions of this study:

1. This work proposes a novel end-to-end hybrid deep learning architecture that improves MI-EEG decoding performance by combining the local feature extraction of multi-scale CNNs with the global dependency modeling of the Transformer’s self-attention mechanism, capturing both detailed local features and broader dependencies.
2. This work outperforms multiple state-of-the-art (SOTA) methods in decoding performance on the BCI Competition IV-2a and IV-2b datasets, demonstrating its potential as a new benchmark for EEG decoding.
3. Comprehensive experiments were conducted to examine the impact of the hyperparameters in the convolution and Transformer modules, as well as data augmentation.
4. To promote reproducibility and support further research, the MSCFormer source code has been made publicly available at https://github.com/snailpt/MSCFormer.

The rest of this paper is organized as follows: Sect. 2 provides a comprehensive review of related work. Section 3 introduces the datasets, data preprocessing methods, and data augmentation techniques, followed by a detailed description of the proposed model architecture. Section 4 evaluates the performance of the model through extensive experimentation. Section 5 discusses our main findings, and finally, conclusions are drawn.

## Related work

In this section, we describe the key techniques involved in our proposed method, including multi-scale CNN (MSCNN)-based approaches and Transformer-based networks. A comparative summary of representative works is provided in Table 1.

### MSCNN-based approaches

Over the past decade, CNNs have achieved remarkable success in computer vision, largely due to their ability to autonomously learn both local and global features through convolution operations. When processing EEG sequence signals, convolution operations are also effective in capturing temporal and spatial features, which are critical for decoding brain signals. Consequently, CNNs have been widely applied in BCI^31,32^. CNN-based MI-EEG decoding methods encompass both SSCNNs and MSCNNs. SSCNNs extract features from EEG signals using a single temporal and spatial convolution scale. However, MI-EEG signals inherently exhibit complex spatiotemporal patterns and multi-band frequency characteristics, which cannot be fully captured by a fixed-scale temporal convolution. Moreover, due to significant inter-subject variability in EEG signals, the optimal convolution scale often varies across individuals.

To address these limitations, MSCNNs perform convolutions across multiple scales, allowing for a more comprehensive capture of the complex characteristics in EEG signals and improving classification performance. For example, Amin et al.^33^ proposed MCNN, a multi-layer CNN fusion method that integrates four parallel CNN streams with varying depths and kernel sizes. By leveraging transfer learning and feature fusion, MCNN enhances MI-EEG classification accuracy by capturing diverse spatiotemporal patterns in EEG signals. Dai et al.^34^ proposed HS-CNN, which decomposes raw EEG signals into $\:\theta\:$, $\:\mu\:$, and $\:\beta\:$bands to address inter-subject variability, and employs hybrid-scale convolution kernels (1 × 45, 1 × 65, 1 × 85) combined with a time-frequency data augmentation method to achieve SOTA classification accuracy. However, the large parameter count (> 420 K per filter band) restricts the system’s applicability. Jia et al.^35^ developed a multi-branch multi-scale CNN (MMCNN) for MI-EEG classification, using five parallel EEG Inception Networks (EINs) with varying kernel scales to capture diverse frequency information, and the squeeze-and-excitation (SE) attention mechanism to enhance performance by reweighting channel features. Altuwaijri and Muhammad^36^ proposed MBEEGNet, composed of multiple EEGNets with different configurations, and MBShallowConvNet, made up of multiple distinct ShallowConvNets. Their multi-branch structure allows for more comprehensive EEG signal feature extraction, overcoming the single-scale limitation of traditional methods and leading to superior classification performance across multiple datasets. Roy et al.^37^ proposed a multi-scale CNN, which filters EEG signals into $\:\delta\:$, $\:\theta\:$, $\:\alpha\:$, and $\:\beta\:$ bands, applying multi-scale convolution blocks with varying kernel sizes to each band. The model also incorporates user-specific features such as differential entropy (DE) and neural power spectrum (NPS), further enhancing performance.

### Transformer-based networks

While MSCNNs capture more information than SSCNNs, their limited receptive field restricts the modeling of long-term dependencies, limiting further improvements in MI-EEG decoding. In contrast, the self-attention mechanism in the Transformer architecture, with its global receptive field, effectively captures global dependencies and enhances decoding performance. Song et al.^38^ proposed the Spatial-Temporal Tiny Transformer (S3T) for EEG decoding, addressing the limitations of CNNs in capturing global dependencies. Their model applies spatial filtering before utilizing self-attention along the feature channel and temporal dimensions to enhance relevant features. Tao et al.^39^ proposed the Gated Transformer, a family of Transformer models incorporating various gating mechanisms to enhance EEG classification. By replacing standard residual connections with different gating mechanisms, their approach stabilizes training and improves long-term dependency modeling in EEG sequences. Xie et al.^29^ proposed a Transformer-based deep learning framework for MI-EEG classification, incorporating both spatial and temporal dependencies. Their study introduced five Transformer-based models, explored three types of positional embeddings, and achieved SOTA accuracy on the PhysioNet EEG Motor Imagery Dataset. Song et al.^40^ proposed EEG Conformer, a Convolutional Transformer model for unified EEG decoding that integrates CNN-based local feature extraction with Transformer-based global feature learning. Inspired by the Conformer model, Zhao et al.^41^ proposed CTNet, which integrates a single-scale CNN module similar to EEGNet for local feature extraction and incorporates a Transformer for global feature modeling, leading to enhanced classification performance.

Additionally, some researchers have begun exploring EEG decoding methods that integrate multi-scale CNNs with the self-attention mechanism. Ahn et al.^42^ proposed MS-TSformer-DS, a hybrid EEG decoding model that combines multi-scale convolutional blocks, temporal-spatial Transformer encoders, and a dual-stream spatial learner to enhance spatial feature representation. Their model demonstrated strong performance across multiple EEG datasets, including a private dataset, BCI Competition IV-2a, and the Arizona State University (ASU) dataset. Tao et al.^43^ proposed ADFCNN, an attention-based dual-scale fusion CNN for MI-EEG classification. It employs dual-scale temporal and spatial convolutions to extract spectral-spatial features, while a self-attention mechanism enhances feature fusion by capturing cross-scale dependencies.

Building on these previous studies, our research makes further contributions to the field. Inspired by these prior studies, we propose MSCFormer as an effective solution for MI-EEG decoding.

**Table 1:** Summary of related work.

Related work	Methods	Database	Accuracy %	Comment
Amin et al. 2019^33^	MCNN	BCI IV-2a High Gamma Dataset	75.7 95.4	Multi-branch CNN with varying depths/kernels, using transfer learning and feature fusion.
Dai et al. 2019^34^	HS-CNN	BCI IV-2a BCI IV-2b	91.57 ± 5.41 87.64 ± 8.00	Hybrid-scale CNN decomposing EEG into θ, µ, and β bands with time-frequency data augmentation.
Jia et al. 2020^35^	MMCNN	BCI IV-2a BCI IV-2b	81.4 ± 11.7 84.4 ± 7.5	Multi-branch CNN with varying kernel sizes and SE attention.
Altuwaijri and Muhammad 2022^36^	MBShallowConvNet / MBEEGNet	BCI IV-2a High Gamma Dataset	81.15 ± 9.04 95.11 ± 4.62 / 82.01 ± 10.13 95.30 ± 3.50	Extensions of ShallowConvNet and EEGNet, respectively, incorporating three CNN branches with different configurations to enhance multi-scale feature extraction.
Roy et al. 2022^37^	MS-CNN	BCI IV-2b	93.74 ± 2.80	Utilizes four-band decomposition, multi-scale convolution, and user-specific DE and NPS features for feature extraction.
Song et al. 2021^38^	S3T	BCI IV-2a BCI IV-2b	82.59 ± 12.52 84.26 ± 10.03	A Transformer-based method with spatial filtering and self-attention for spatiotemporal feature learning.
Tao et al. 2021^39^	GRUGate Transformer	Brain-Visual Dataset PhysioNet	61.96 ± 10.09 55.40 ± 2.09	A Transformer-based method with gating mechanisms to enhance stability and long-term feature extraction.
Xie et al. 2022^29^	s-Trans / t-Trans / s-CTrans / t-CTrans / f-CTrans	PhysioNet	Best accuracy: 83.31 (2-class), 74.44 (3-class), 64.22 (4-class)	Proposed five Transformer-CNN hybrid models integrating spatiotemporal dependencies with optimized positional embeddings.
Song et al. 2023^40^	Conformer	BCI IV-2a BCI IV-2b SEED	78.66 ± 14.43 84.63 ± 11.49 95.30	A Convolutional Transformer model integrating CNN for local feature extraction and Transformer for global dependency modeling.
Zhao et al. 2024^41^	CTNet	BCI IV-2a BCI IV-2b	82.52 ± 9.61 88.49 ± 9.03	A hybrid CNN-Transformer model for MI-EEG classification, enhancing spatiotemporal representation learning.
Ahn et al. 2023^42^	MS-TSformer-DS	Private EEG BCI IV-2a ASU	62 ± 6 70 ± 9 70 ± 7	A hybrid CNN-Transformer model integrating multi-scale temporal convolution, temporal-spatial Transformer, and dual-stream spatial learning.
Tao et al. 2024^43^	ADFCNN	BCI IV-2a BCI IV-2b OpenBMI	79.39 ± 10.23 87.81 ± 8.40 65.26 ± 13.50	A dual-scale CNN integrating self-attention for enhanced spectral-spatial fusion.

## Materials and methods

### Datasets

To evaluate the effectiveness of our proposed model, we used two publicly available benchmark datasets: BCI IV-2a and IV-2b. Detailed descriptions of these datasets, preprocessing, and data augmentation are provided below.

1. BCI IV-2a dataset (2a): The dataset comprises EEG recordings from nine subjects (A01-A09), each engaged in four distinct motor imagery tasks: imagining movements of the left-hand, right-hand, both feet and tongue. Each subject participated in two recording sessions conducted on different days, yielding a total of 288 trials per session. The EEG recordings were obtained using 22 electrodes, with a sampling rate of 250 Hz, and each recording lasted for 7 s. In our experiments, we utilized the temporal segment from 2 to 6 s. Each trial was represented as a matrix of dimensions (22, 1000).
2. BCI IV-2b dataset (2b): The dataset comprises EEG recordings from nine right-handed subjects (B01-B09) over five sessions, with about 720 trials per subject. The first two sessions lack feedback, whereas the subsequent three sessions provide feedback. Each session includes multiple runs in which subjects imagine left- or right-hand movements. The EEG recordings were captured using three bipolar channels at a sampling frequency of 250 Hz. In our experiments, we utilized the temporal segment from 3 to 7 s. Each trial was represented as a matrix with dimensions (3, 1000).
3. Data Preprocessing: The raw EEG recordings are defined as $\:\left\{\right({X}_{i},{y}_{i}\left)\right|i=\text{1,2},\ldots,N\}$, where $\:{X}_{i}\in\:R$^*C*×*T*^ represents the *i*-th trial consisting of *C* channels and *T* sampling time points, $\:{y}_{i}$ is the sample label corresponding to $\:{X}_{i}$, and *N* is the total number of trials. In this study, we employed a zero-mean standardization (STD) method for preprocessing the EEG recordings. Notably, we did not apply any band-pass filtering or artifact removal techniques. The zero-mean standardization method was used to reduce the influence of signal amplitude variations and enhance the robustness of signal processing and classification algorithms by ensuring that the data were on a consistent scale. The calculation method is expressed as follows:
  1 $$\:\stackrel{\sim}{{X}_{i}}=\frac{{X}_{i}-\mu\:}{\sigma\:}$$
  where $\:\stackrel{\sim}{{X}_{i}}$ is the normalized EEG signal. $\:\mu\:$ and $\:\sigma\:$ denote the mean and standard deviation (S.D.) of the raw EEG data, respectively, calculated using the training dataset and then applied directly to the test dataset.
4. Data Augmentation: Given the stringent criteria for participant recruitment and the complexities of experimental setups, it is inherently challenging to acquire substantial, high-quality EEG data. DL models trained on such small datasets are particularly susceptible to overfitting. Therefore, implementing data augmentation techniques to enhance the generalizability and robustness of MI-EEG models is essential. We employ the segmentation and reconstruction (S&R) method^44^ in the time domain to augment training data, which involves a systematic approach to artificially increasing the amount and variability of EEG training datasets. The S&R method includes two steps: segmentation and reconstruction. The workflow of the S&R method is shown in Fig. 1. In the segmentation phase, each EEG trial is divided into *N*_*s*_ segments based on time intervals, ensuring that each segment captures a subset of the entire signal’s timeframe. As shown in Fig. 1, *N*_*s*_ equals 3, indicating each EEG trial is segmented into three parts, labeled as A, B, and C, respectively. In the reconstruction phase, new artificial EEG trials are generated by randomly recombining these segments in a manner that aligns with the natural progression of time within EEG recordings. The number of training samples augmented is denoted as *N*_*A*_. This approach not only increases data diversity by mixing segments from various trials, introducing new patterns for the model to learn but also maintaining the temporal structure of the EEG signals, which is crucial for retaining the physiological relevance of the EEG data.
  **Fig. 1:**
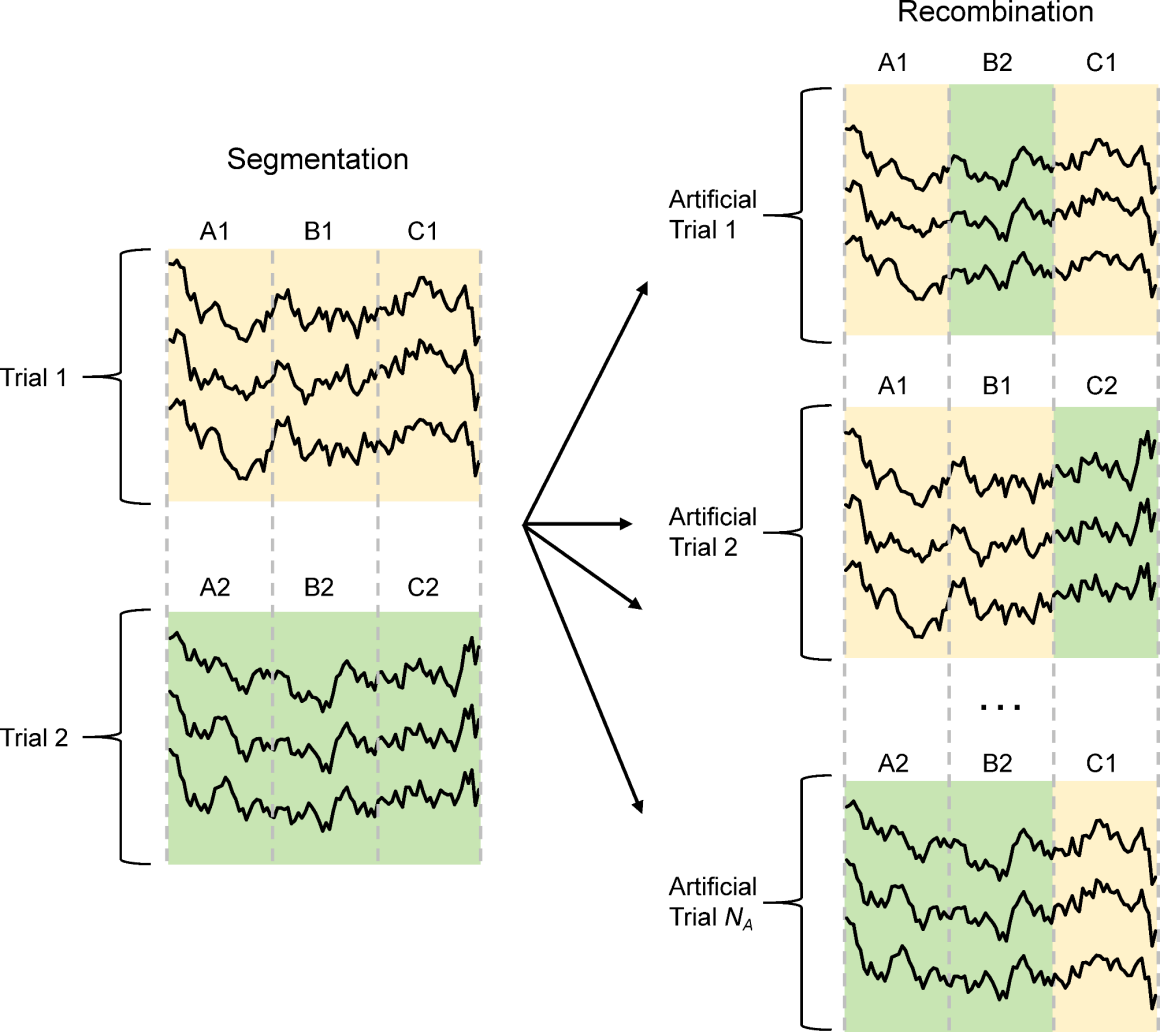
Principle of the S&R data augmentation method in time domain.

### Overview of the proposed framework

**Fig. 2:**
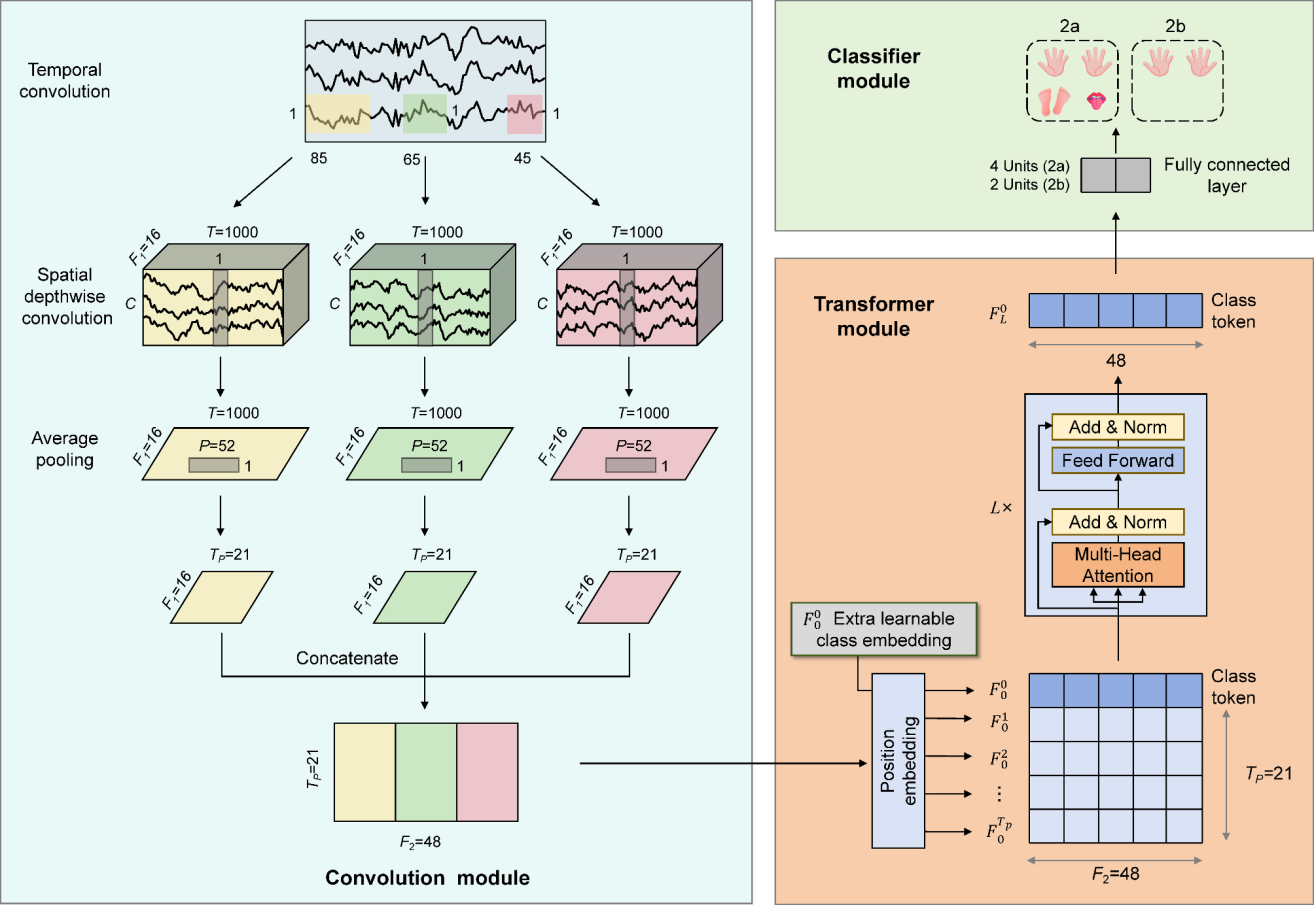
The framework of proposed MSCFormer, including a convolution module, a Transformer module, and a classifier module.

CNNs are effective at capturing local features, while Transformer networks excel at modeling global dependencies. In this paper, we introduce MSCFormer, an end-to-end MI-EEG classification framework that integrates CNNs and the Transformer encoder to leverage both architectures’ strengths. The overall framework of MSCFormer is depicted in Fig. 2. MSCFormer first employs a convolution module to extract spatiotemporal features at various scales from the EEG data. The Transformer module then applies a multi-head self-attention mechanism (MHA) to model global dependencies across these multi-scale features, dynamically emphasizing the most relevant features for classification. Finally, a fully connected layer classifies the extracted features into distinct categories, completing the MI-EEG classification process.

### Convolution module

The convolution module includes three CNN branches, each similar to the Shallow ConvNet proposed in^20^. However, each branch uses spatial depthwise convolution instead of spatial standard convolution, which has demonstrated better performance. Additionally, each branch employs different hyperparameters compared to those in^20^. The distinction between the three branches lies in the different kernel sizes used for temporal convolution, enabling the learning of EEG features at various time scales.

Each branch in the convolution module comprises a temporal convolution layer, a spatial depthwise convolution layer, and an average pooling layer, as illustrated in Fig. 2. The temporal convolution layer utilizes *F*_1_ temporal filters with a kernel size of (1, *K*_*c*_). Different values of *K*_*c*_ can capture EEG temporal features at various scales. Following the setup in reference^34^, *K*_*c*_ values for the three CNN branches are set to 85, 65, and 45, respectively. The spatial depthwise convolution layer independently applies spatial convolutions to each temporal feature map, effectively learning spatial filters associated with specific frequency bands. Additionally, it reduces the number of training parameters, lowering model complexity and computational resource requirements. The spatial convolution kernel size is (*C*, 1), producing *F*_1_ feature maps that integrate both temporal and spatial features with a shape of (*F*_1_, *T*). Following the spatial convolution, a batch normalization (BN) layer is applied, stabilizing the data distribution and facilitating smoother gradient flow, thereby improving training efficiency and effectiveness. Subsequently, an exponential linear unit (ELU) activation function is applied. An average pooling layer with a kernel size of (1, *P*) is then used. This pooling step not only reduces the feature map dimensions but also smooths the spatiotemporal feature maps, reducing local noise and aiding the model in learning global features more effectively. Each branch applies a dropout operation with a probability of 0.5 after the average pooling layer to reduce overfitting. Therefore, the feature dimension output by each CNN branch is (*F*_1_, *T*_*p*_), where *T*_*p*_ represents the length of the feature series and is given by *T* divided by *P*. Next, the feature maps obtained from the three CNN branches are transposed by swapping the convolution feature channel dimension with the time dimension. These feature maps are then concatenated along the feature channel dimension, resulting in a fused feature map *X*_*F*_ with dimensions (*T*_*p*_, *F*_2_), where *F*_2_ is equal to three times *F*_1_. In this way, the fused feature maps at each temporal point are fed as tokens into the subsequent Transformer module.

### Transformer module

To further enhance the features extracted by the CNN module, we employ a Transformer module to model the global dependencies of the multi-scale MI-EEG features. The self-attention mechanism in the Transformer module provides a global receptive field, enabling the model to capture long-range dependencies. It dynamically prioritizes the most relevant features for classification, ensuring the model focuses on the most informative aspects. To quantify the attention allocated by the model to different feature channels, we introduce an additional learnable vector ($\:{F}_{0}^{0}$) as a class token, similar to the class token used in BERT^45^, appended to the front of the feature maps. Subsequently, the learnable position embedding *F*_*pos*_ are added to the sequence of features to retain positional information. Therefore, the feature embedding *F*_0_ serves as the input to the Transformer encoder, where *F*_0_ is calculated as follows.

2 $$\:{F}_{0}=[{F}_{0}^{0},\:{X}_{F}]\:+\:{F}_{pos}$$

The features are then encoded using an *L*-layer deep Transformer encoder, where the class token ($\:{F}_{L}^{0}$) from the *L*-th layer represents the output of the Transformer module, serving as the MI-EEG’s feature representation.

**Fig. 3:**
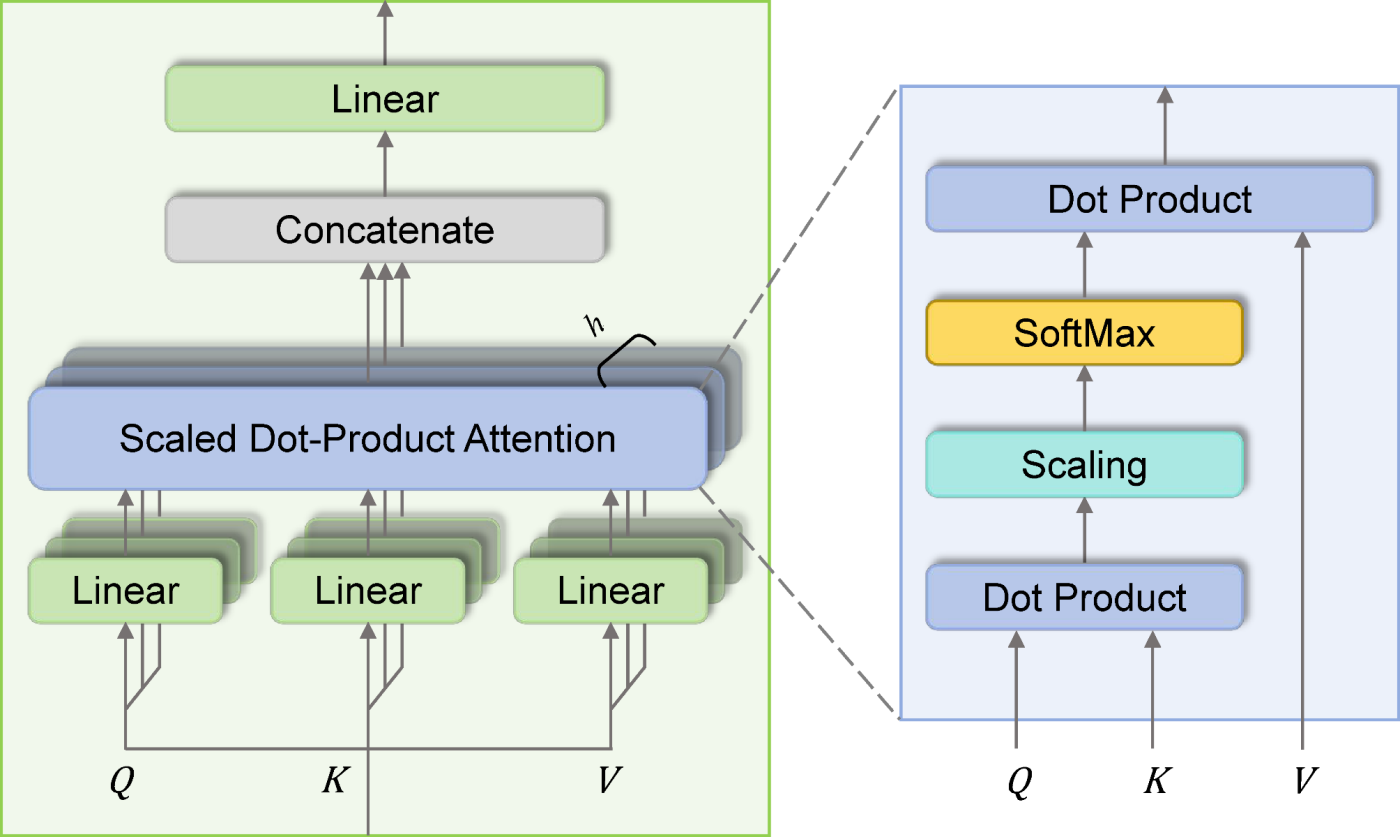
Multi-head self-attention.

The Transformer encoder consists of MHA and feed forward (FF) blocks. MHA comprises multiple self-attention layers, known as heads, which implement scaled dot-product attention, as illustrated in Fig. 3. Each self-attention layer consists of three main components: query $\:Q$, keys $\:K$, and values $\:V$. $\:Q$, $\:K$, and $\:V$ are computed from the input features *F* through linear transformations. Specifically, $\:{Q}_{i}$, $\:{K}_{i}$, and $\:{V}_{i}$ at the *i*-th head of the self-attention layer are calculated using the following formulas :

3 $$\:{Q}_{i}={F}_{i}{W}_{i}^{Q}$$

4 $$\:{K}_{i}={F}_{i}{W}_{i}^{K}$$

5 $$\:{V}_{i}={F}_{i}{W}_{i}^{V}$$

Where $\:{W}_{i}^{Q}$, $\:{W}_{i}^{K}$ and $\:{W}_{i}^{V}$ are learnable parameters in the linear transformation. The self-attention scores are calculated using the dot product of the $\:Q$ and $\:K$ matrices, scaled by the square root of the dimension of the $\:K$ (*d*_*k*_) to prevent the scores from becoming too large:

6 $$\:\text{S}\text{A}({Q}_{i},\:{K}_{i}{,V}_{i})=\text{s}\text{o}\text{f}\text{t}\text{m}\text{a}\text{x}\left(\frac{{Q}_{i}{K}_{i}^{T}}{\sqrt{{d}_{k}}}\right){V}_{i}$$

The softmax function is applied to normalize these scores into probabilities. Finally, these probabilities are used to perform a weighted sum of the value vectors. MHA enables the model to focus simultaneously on information from different representation subspaces at various locations, enhancing its ability to capture complex patterns and relationships among EEG features. It executes multiple self-attention operations in parallel and then projects its concatenated output.

7 $$\:\text{M}\text{H}\text{A}(Q,\:K,\:V)=\text{C}\text{o}\text{n}\text{c}\text{a}\text{t}\left(\text{S}\text{A}\right({Q}_{1},{K}_{1},{V}_{1}),\:\ldots,\text{S}\text{A}({Q}_{h},{K}_{h},{V}_{h}\left)\right){W}^{O}$$

where *W*^*O*^ is a learnable weight matrix. The output of the MHA block is typically followed by a residual connection and layer normalization (LN):

$$\:O_{{MHA}} = {\text{LN}}\left( {{\text{MHA}}\left( {Q,\:K,\:V} \right) + F} \right).$$

The FF block consists of two linear transformations with a Gaussian error linear unit (GELU) activation function in between:

9 $$\:\text{F}\text{F}\text{N}\left({O}_{MHA}\right)=\text{G}\text{E}\text{L}\text{U}({O}_{MHA}{W}_{1}+{b}_{1}{)W}_{2}+{b}_{2}$$

where *W*_1_ and *W*_2_ are weight matrices, and *b*_1_ and *b*_2_ are bias terms. Similar to the MHA block, the output of the FF block is accompanied by a residual connection and followed by LN:

$$O\, = \,{\text{LN}}\left( {{\text{FFN}}\left( {O_{{MHA}} } \right) + O_{{MHA}} } \right).$$

Therefore, the MI-EEG feature representation $\:{F}_{L}^{0}$ in *F*_*L*_ serves as the output of the Transformer module and also acts as the input to the classifier module.

### Classifier module

The classifier module consists of a fully connected layer with a softmax function, where the number of neurons *M* is set to match the number of classes in the classification task. To reduce overfitting, dropout is applied to the input features before classification, with the dropout rate set at 0.25. The cross-entropy loss is employed as the loss function for model training, which is expressed as:

11 $$\:\text{L}\text{o}\text{s}\text{s}(y,\:\widehat{y})=-\frac{1}{N}\sum\:_{i=1}^{N}\sum\:_{c=1}^{M}y\text{l}\text{o}\text{g}\left(\widehat{y}\right)$$

where *y* represents the actual labels, and $\:\widehat{y}$ represents the predicted labels.

### Performance metrics

We employ the most commonly used metrics, accuracy and kappa, for the evaluation of the MI-EEG classification method. Accuracy is defined as follows:

12 $$\:\text{A}\text{c}\text{c}\text{u}\text{r}\text{a}\text{c}\text{y}\:=\frac{TP+TN}{TP+TN+FP+FN}$$

where *TP* and *TN* represent true positives and true negatives, respectively, while *FP* and *FN* represent false positives and false negatives. The kappa coefficient is a normalized measure that takes into account the chance level and is defined as follows:

13 $$\:kappa\:=\:\frac{{p}_{o}-{p}_{e}}{1-{p}_{e}}$$

where $\:{p}_{o}$ denotes the observed accuracy (the average accuracy across all the trials) and $\:{p}_{e}$ denotes the expected accuracy (the accuracy of a random guess). Generally, the higher the accuracy and kappa, the better the model’s classification performance. The Wilcoxon signed-rank test is used to assess statistical significance. A p-value greater than 0.05 indicates the absence of a statistically significant difference. Conversely, a p-value less than 0.05 (denoted as ‘*’) signifies a significant difference, while a p-value less than 0.01 (denoted as ‘**’) indicates a highly significant difference.

## Experiments and results

### Experiment settings

Our method is implemented in PyTorch and utilizes an Intel Core i9-9820X CPU and an NVIDIA RTX 4090 GPU. We classify the BCI IV-2a and IV-2b datasets using only EEG channel data, entirely discarding the three EOG channels in our experiments. We conducted subject-specific classification experiments and adhered to the data division scheme outlined in the competition guidelines. For the BCI IV-2a dataset, we used the first session as the training set and the second session as the test set. For the BCI IV-2b dataset, we used the first three sessions as the training set and the last two sessions as the test set. To evaluate the stability and generalization ability of our model, we performed five-fold cross-validation (CV) on the original training set. We divided the original training set into five approximately equal subsets. Then, we used one subset as the validation set and combined the remaining four subsets with the S&R augmented dataset for training. We selected the model with the lowest loss on the validation set as the best model and tested it on the test set. This process was repeated for each of the five original training subsets, and the final performance metric was obtained by averaging the results on the test set.

During S&R data augmentation, each EEG trial is segmented into eight segments (*N*_*s*_ = 8). We use the Adam optimizer to train the model, with the learning rate, *β*_1_, and *β*_2_ set to 0.001, 0.5, and 0.999, respectively. The batch size and number of epochs for training are set to 288 and 1000, respectively. These training hyperparameter settings are adopted based on the guidelines in^41^. Given the fewer electrode channels in the BCI IV-2b dataset, we applied L2 regularization to reduce overfitting, with the weight decay parameter set to 0.001. The three convolutional kernel sizes in the convolution module were adopted from^34^, while the remaining hyperparameters of the MSCFormer architecture were determined through extensive experiments. Unless specified otherwise, the hyperparameters of the MSCFormer architecture are detailed in Table 2.

**Table 2:**
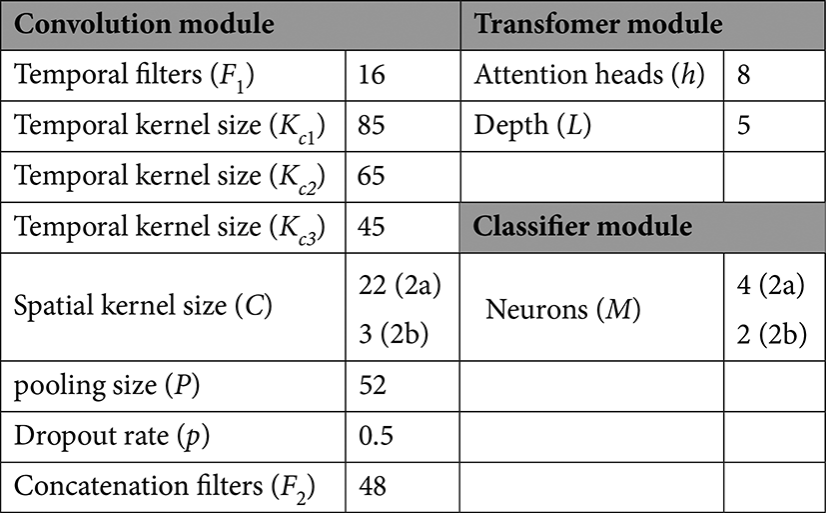
The hyperparameters of MSCFormer architecture.

### Ablation study

To systematically examine the impact of the Transformer module, data augmentation, and temporal convolution kernel sizes within the MSCFormer framework, we conducted a series of rigorous ablation studies on the BCI IV-2a and IV-2b datasets. The ablation study was conducted using five distinct experimental configurations: (1) the fully integrated MSCFormer model, (2) the model without the Transformer module (w/o Trans), in which multi-scale CNN features are concatenated, flattened, and fed into the classifier module, (3) the model without data augmentation (w/o Aug), (4) the model lacking both the Transformer and data augmentation (w/o Trans & Aug), and (5) the fully integrated model with smaller convolutional kernels (w/ Small-K), which utilizes reduced temporal convolution kernel sizes (64, 32, 16), as adopted in^22,36^.

**Fig. 4:**
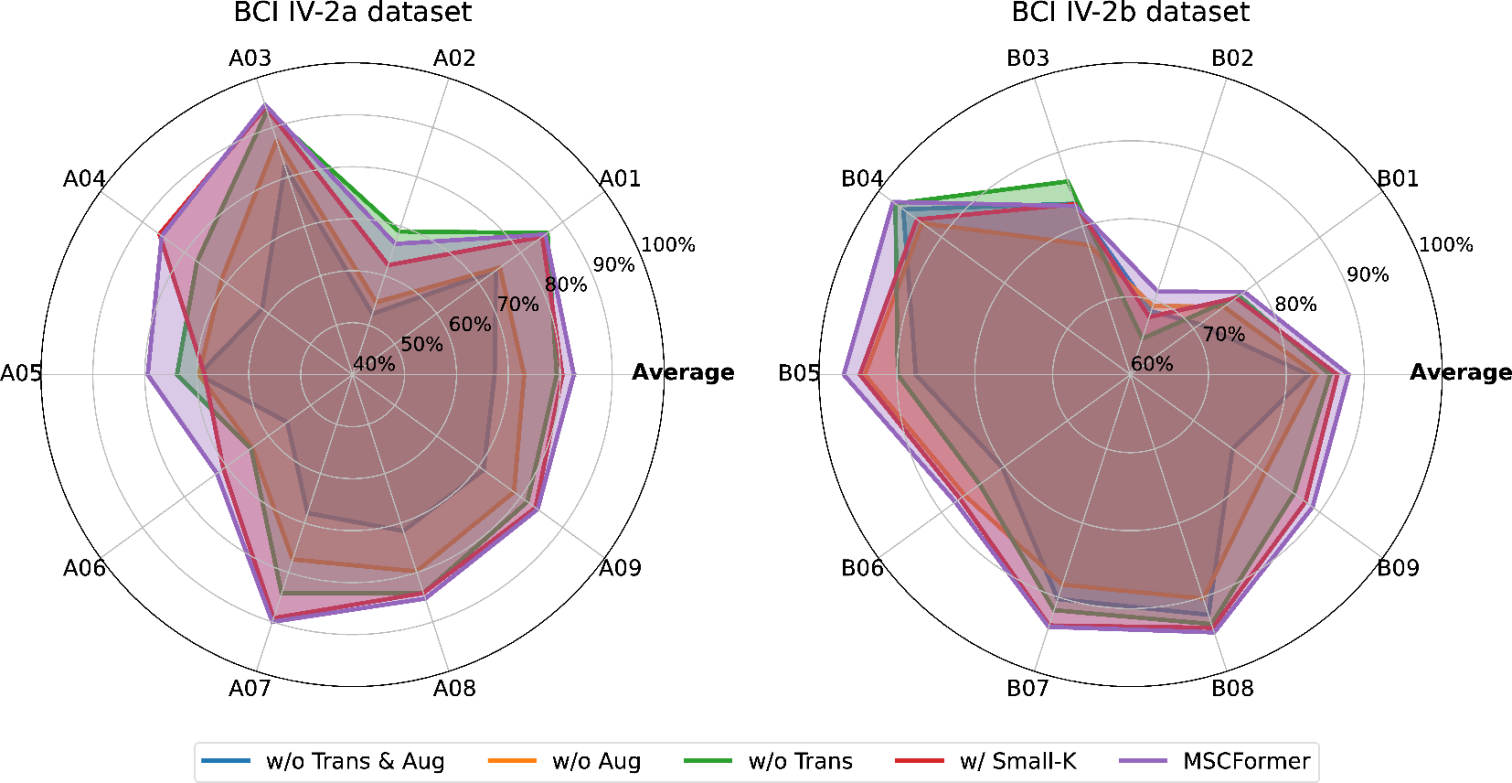
Radar chart visualization of ablation effects on average accuracy.

Figure 4 illustrates the average decoding accuracies for each subject obtained through a five-fold CV across the specified configurations. MSCFormer consistently achieved the highest average decoding accuracies on both datasets, reaching 82.60% on the BCI IV-2a dataset and 88.00% on the BCI IV-2b dataset. Removing the Transformer module resulted in a 3.30% decrease in average accuracy on the BCI IV-2a dataset (*p* = 0.055), with particularly substantial drops observed in subjects A04, A05, A06, and A07, where accuracies decreased by 8.40%, 5.63%, 8.13%, and 5.76%, respectively. On the BCI IV-2b dataset, removing the Transformer led to a notable decrease in average accuracy of 2.38% (*p* < 0.05). Without data augmentation, removing the Transformer module reduced the average classification accuracy by 5.69% (*p* < 0.01) on the BCI IV-2a dataset and by 1.06% on the BCI IV-2b dataset. Removing data augmentation alone led to significant decreases in recognition accuracy on both the BCI IV-2a and BCI IV-2b datasets, with significant reductions of 9.57% (*p* < 0.01) and 4.12% (*p* < 0.01), respectively. Simultaneously removing the Transformer module and data augmentation led to significant decreases in average recognition accuracy: 15.26% (*p* < 0.01) on the BCI IV-2a dataset and 5.18% (*p* < 0.01) on the BCI IV-2b dataset. These results demonstrate the critical role of the Transformer module and data augmentation in enhancing the decoding accuracy of the MSCFormer model. Furthermore, using smaller convolutional kernels in the convolution module led to a significant decrease in average classification accuracy, with a drop of 2.21% (*p* < 0.05) on BCI IV-2a and 1.45% (*p* < 0.01) on BCI IV-2b.

**Fig. 5:**
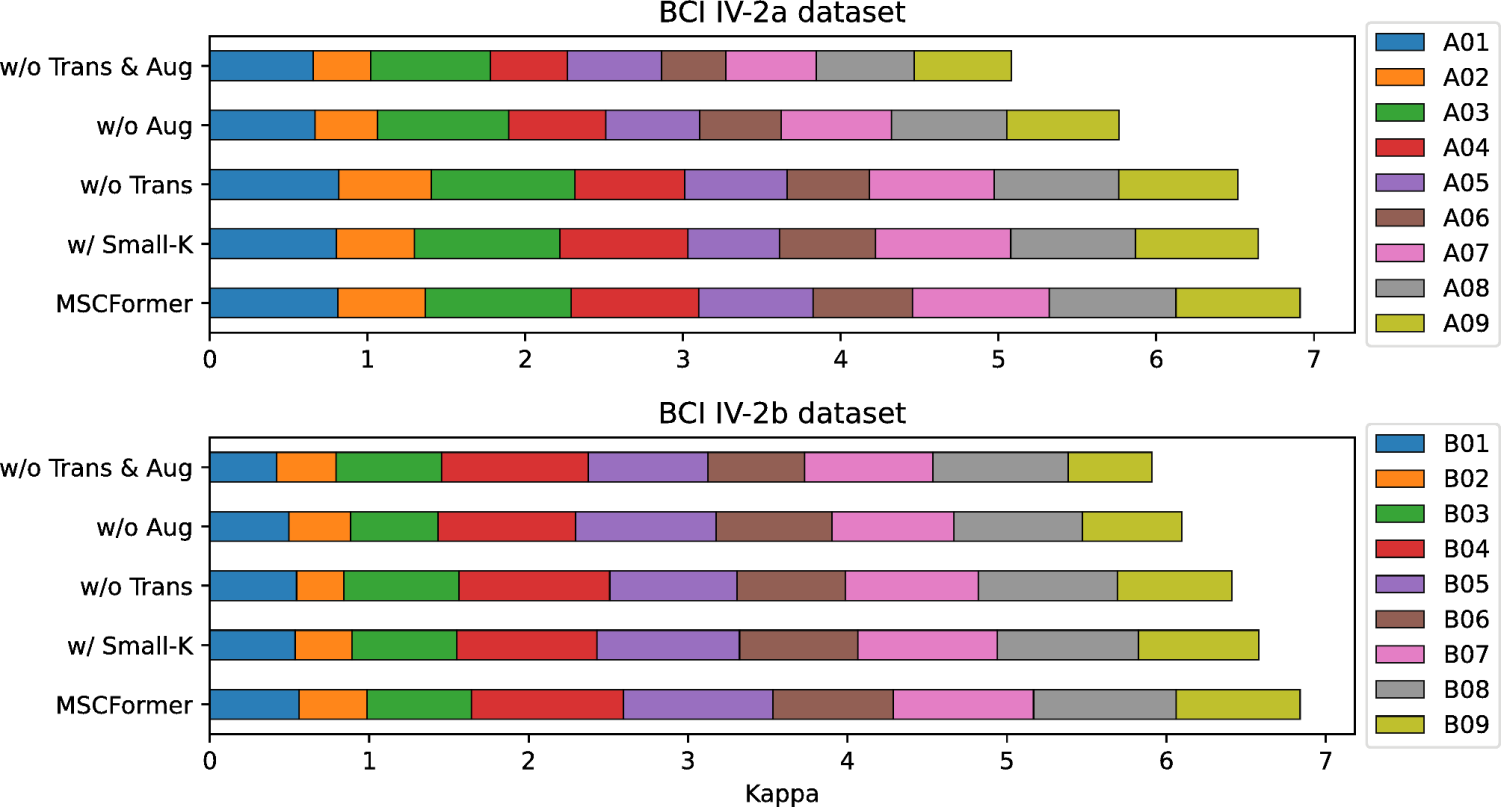
Stacked bar chart of ablation study on average kappa coefficients.

Figure 5 provides a comparative exposition of kappa coefficients across the various experimental conditions for each subject. The comprehensive MSCFormer setup outperformed all other conditions across both datasets, achieving the highest cumulative kappa. This result highlights the essential roles of both the Transformer and data augmentation in enhancing the model’s robustness and consistency. Additionally, the use of larger convolutional kernels yielded superior performance.

### Impact of the depth of transformer

**Fig. 6:**
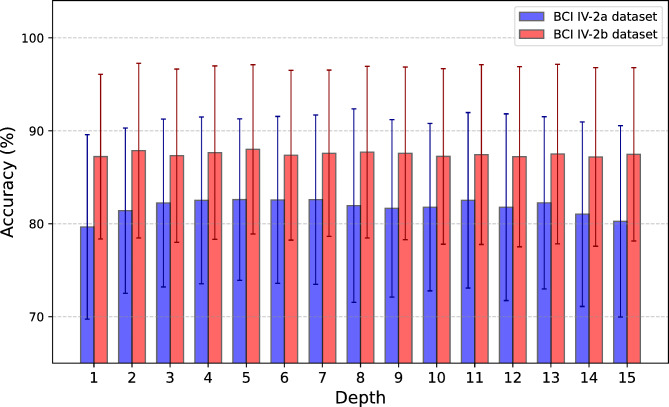
The impact of Transformer encoder depth on accuracy.

Typically, within the Transformer encoder module, the depth *L* of the Transformer significantly influences model performance. Figure 6 depicts the evolution of recognition accuracy with increasing depths. On the BCI IV-2a dataset, the model achieves the highest average accuracy at a depth of 5, which is 2.94% higher than at depth 1 (*p* < 0.05). Beyond this, further increasing the Transformer depth results in a decline in accuracy. Similarly, on the BCI IV-2b dataset, the average accuracy at depth 5 is notably improved by 0.68% compared to depth 3 (*p* < 0.05). These findings suggest that while increasing depth improves the model’s ability to capture complexity, it also raises the risk of overfitting if the training dataset does not scale accordingly.

### Impact of the number of heads in MHA

**Fig. 7:**
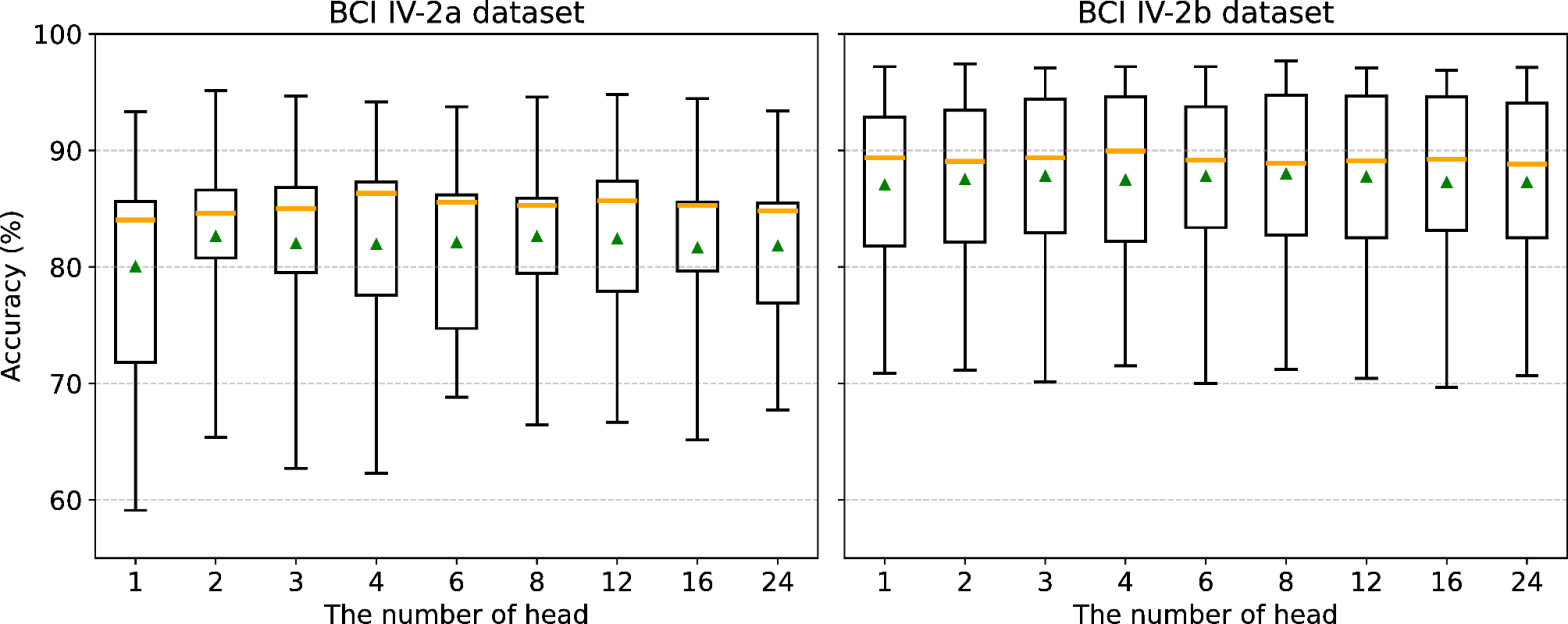
The impact of the number of heads in MHA on model accuracy across datasets. The orange line within each box represents the median, while the green triangle indicates the mean.

In Transformer models, the number of heads in the MHA mechanism is a key parameter that helps in learning different aspects of features. We assessed the impact of varying the number of heads on MSCFormer’s performance, with the results shown in Fig. 7. The MSCFormer model with a single head had the lowest average classification accuracy on both datasets. For the BCI IV-2a dataset, the eight-head model achieved 2.58% higher accuracy than the single-head model (*p* < 0.05). On the BCI IV-2b dataset, the eight-head model outperformed the single-head model by 0.96% (*p* < 0.05) and the 24-head model by 0.75% (*p* < 0.01). These results suggest that appropriately increasing the number of heads can significantly improve accuracy. Additionally, performance on the BCI IV-2a dataset showed greater fluctuation compared to BCI IV-2b, possibly indicating that the model’s sensitivity to the number of heads increases with task complexity.

### Impact of the pooling size

**Fig. 8:**
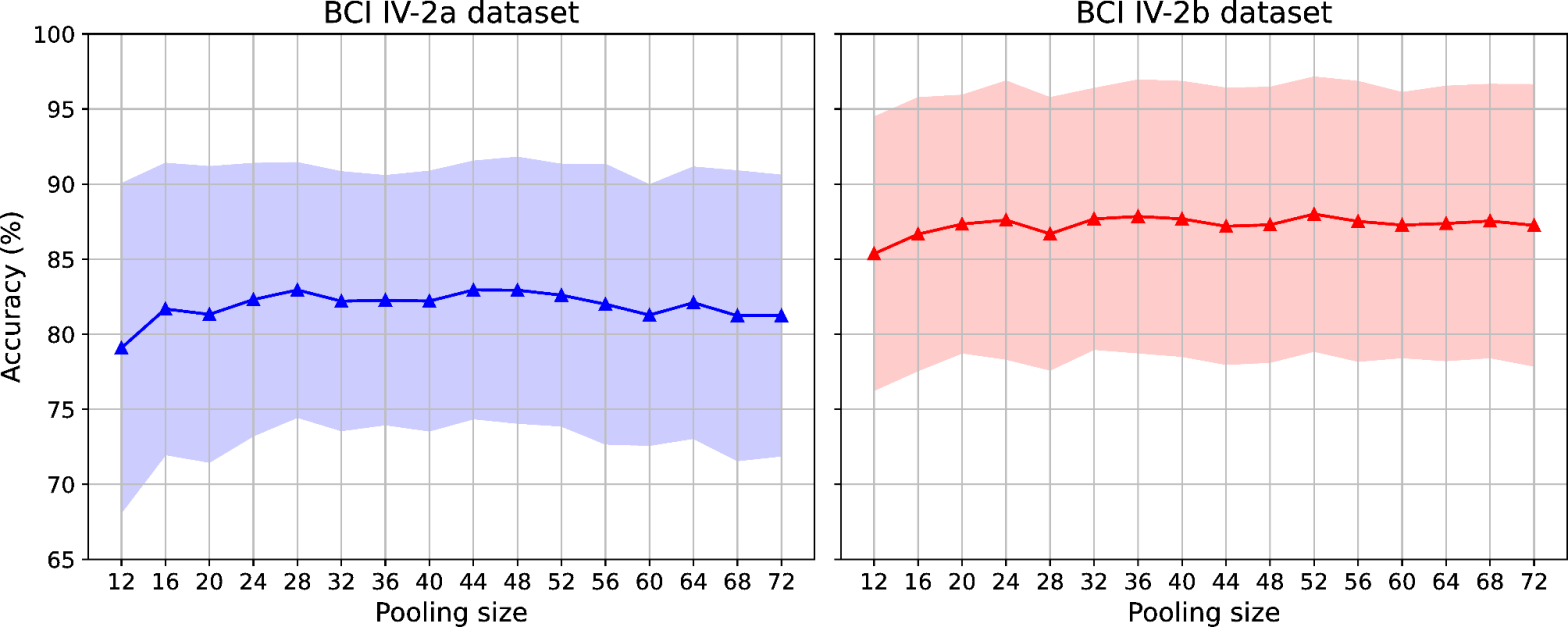
The impact of the pooling size on the accuracy for different datasets.

In our study, we explored the impact of varying pooling sizes on the performance of MSCFormer by adjusting the pooling size from 12 to 72, with increments of 4, resulting in corresponding token lengths ranging from 84 to 14. As depicted in Fig. 8, MSCFormer exhibited an increasing trend in accuracy followed by a decline across the BCI IV-2a and IV-2b datasets. Notably, on the BCI IV-2a dataset, two distinct peaks in classification accuracy were observed at pooling sizes of 28 and 44, both reaching an optimum of 82.95%. In contrast, on the BCI IV-2b dataset, the highest average classification accuracy of 88.00% was achieved at a pooling size of 52. Compared to the lowest performance observed at a pooling size of 12, these peaks represent improvements of 3.87% (*p* < 0.01) and 2.64% (*p* < 0.01) on the BCI IV-2a and IV-2b datasets, respectively. These findings suggest that pooling size should be carefully selected based on the specific characteristics of the dataset.

### Comparison of MSCFormer with SOTA methods

To comprehensively assess our model’s performance, we selected several SOTA methods, including three SSCNN-based models (Shallow ConvNet^20^, Deep ConvNet^20^, EEGNet^24^), two MSCNN-based models (MMCNN^35^, MBEEGNet^36^), and two hybrid CNN-Transformer models (Conformer^40^, ADFCNN^43^). Below is a brief introduction to these methods:

- Shallow ConvNet: This model uses a large-kernel temporal convolution layer followed by a spatial convolution layer, applying nonlinear activation and pooling, and concludes with a fully connected layer for classification.
- Deep ConvNet: A more complex architecture for EEG signal decoding, it begins with a spatiotemporal convolution layer, followed by three convolutional blocks, each paired with max-pooling layers, and ends with a fully connected layer for classification.
- EEGNet: A compact architecture designed for EEG signal decoding, utilizing depthwise and separable convolutions to capture spatial and temporal features, effectively reducing the number of model parameters.
- MMCNN: This model consists of five parallel EINs, each comprising an EEG Inception block, a residual block, and a SE block.
- MBEEGNet: An extension of EEGNet, featuring multiple parallel EEGNet branches, each with different filter kernel sizes.
- Conformer: This model integrates Shallow ConvNet with Transformer architecture to capture both local spatiotemporal features and global dependencies in EEG features.
- ADFCNN: It combines large and small convolutional kernels to capture dual-scale features in EEG signals, with a self-attention mechanism that dynamically adjusts feature weights for enhanced performance.

The optimal results for MSCFormer on the BCI IV-2a and IV-2b datasets were compared to the SOTA methods. To further validate the effectiveness of our multi-scale convolution approach, we included MSNet, an ablation model without the Transformer module (w/o Trans), in the comparison.

To ensure a relatively fair comparison, we re-evaluated four representative models, including Shallow ConvNet, Deep ConvNet, EEGNet, and MBEEGNet, whose original experimental conditions in the literature differed significantly from those in our study. In these reimplemented experiments, we applied identical experimental conditions, including the same data preprocessing, data augmentation strategies, CV methods, and training hyperparameters (batch size, learning rate, and epochs). Table 3 presents comparisons of accuracy and kappa between MSNet, MSCFormer, and SOTA methods on the BCI IV-2a. Table 4 presents comparisons for the BCI IV-2b. The data for MMCNN, Conformer, and ADFCNN were obtained from their respective references.

A comparison of these CNN models reveals that our proposed MSCNN-based model (MSNet) achieved the second-highest average classification accuracy on the BCI IV-2a dataset, while also exhibiting the smallest accuracy standard deviation and the highest kappa value. Specifically, its average accuracy increased by 4.08% (*p* < 0.01), 1.84%, 1.47%, and 0.54% compared to Shallow ConvNet, EEGNet, Deep ConvNet, and MMCNN, respectively, but was 2.2% lower than that of MBEEGNet. On the BCI IV-2b dataset, MSNet ranked second in both average accuracy and kappa value, with a relatively small standard deviation. Specifically, its average accuracy was 2.36% (*p* < 0.01), 1.24%, 1.16%, and 0.34% higher than those of Deep ConvNet, MMCNN, MBEEGNet, and Shallow ConvNet, respectively, but 1.77% lower than that of EEGNet. These comparative results strongly demonstrate the effectiveness of MSNet’s multi-scale design in addressing the challenge of individual variability in EEG signals.

Tables 3 and 4 show that MSCFormer achieved the best average classification accuracy and kappa values across both datasets, with relatively small standard deviations compared to the other models. Specifically, on the BCI IV-2a dataset, MSCFormer’s average accuracy was 7.80% (*p* < 0.01), 5.56% (*p* < 0.05), and 5.20% (*p* < 0.05) higher than the SSCNN-based models Shallow ConvNet, EEGNet, and Deep ConvNet, respectively. It also outperformed the MSCNN-based models MBEEGNet, MSNet, and MMCNN by 4.26% (*p* < 0.05), 3.72%, and 1.52%, and exceeded the CNN-Transformer hybrid models Conformer and ADFCNN by 4.29% and 3.56% (*p* < 0.05). On the BCI IV-2b dataset, MSCFormer’s average accuracy was 4.72% (*p* < 0.01), 3.60%, 3.52% (*p* < 0.01), 3.37%, 2.70% (*p* < 0.01), 2.36% (*p* < 0.05), 0.59%, and 0.19% higher than Deep ConvNet, MMCNN, MBEEGNet, Conformer, Shallow ConvNet, MSNet, EEGNet, and ADFCNN, respectively. These comparative results highlight MSCFormer’s superior classification accuracy, consistency, and robustness.

**Table 3:** Comparison of the classification accuracy (%) and kappa on the BCI IV-2a dataset.

Method\Subject		A01	A02	A03	A04	A05	A06	A07	A08	A09	Accuracy	S.D.	Kappa	*p*-value
SSCNN	Shallow ConvNet 2017^20^+	80.97	56.46	91.04	72.29	73.54	61.18	77.71	83.89	79.31	75.15	10.85	0.6687	0.004**
	Deep ConvNet 2017^20^+	81.94	52.85	88.61	76.67	74.44	67.29	**91.04**	82.71	84.24	77.75	11.83	0.7034	0.012*
	EEGNet 2018^24^+	85.56	65.63	92.71	67.64	74.10	58.47	85.21	81.60	85.63	77.39	11.46	0.6986	0.020*
MSCNN	MMCNN 2021^35^	82.10	59.80	92.80	69.00	**87.30**	68.50	89.20	**91.60**	**92.60**	81.43	11.75	0.6260	0.910
	MBEEGNet 2022^36^+	82.85	68.33	92.01	76.39	72.78	65.63	85.97	81.88	82.36	78.69	8.59	0.7158	0.020*
	MSNet (proposed)	86.25	**70.69**	91.67	77.22	75.63	66.94	79.65	83.40	81.60	79.23	**7.65**	0.7230	0.074
Hybrid	Conformer 2023^40^	**88.19**	61.46	93.40	78.13	52.08	65.28	92.36	88.19	88.89	78.66	14.43	0.7155	0.359
	ADFCNN 2024^43^	87.15	61.45	93.75	75.69	75.34	65.27	88.54	82.29	85.06	79.39	10.23	**–**	0.020*
	MSCFormer (proposed)	86.11	65.42	**94.10**	**85.97**	80.42	**74.58**	89.93	84.79	85.21	**82.95**	8.06	**0.7726**	**–**

The bold values indicate the best results. The method marked with plus sign (+) are reimplemented.

**Table 4:** Comparison of the classification accuracy (%) and kappa on the BCI IV-2b dataset.

Method \ Subject		B01	B02	B03	B04	B05	B06	B07	B08	B09	Accuracy	S.D.	Kappa	*p*-value
SSCNN	Shallow ConvNet 2017^20^+	75.94	63.86	83.56	96.44	93.13	85.13	91.19	92.00	86.44	85.30	10.10	0.7059	0.008**
	Deep ConvNet 2017^20^+	74.00	61.71	80.13	94.38	88.63	82.19	90.00	91.88	86.63	83.28	10.27	0.6656	0.004**
	EEGNet 2018^24^+	77.56	68.14	**86.94**	97.44	93.63	87.63	93.38	93.44	88.56	87.41	9.23	0.7482	0.129
MSCNN	MMCNN 2021^35^	**84.90**	70.40	75.50	96.30	92.40	86.30	87.60	84.20	81.80	84.40	**7.47**	0.6870	0.055
	MBEEGNet 2022^36^+	77.06	59.50	82.81	94.94	94.19	82.69	91.19	92.75	85.19	84.48	11.19	0.6896	0.008**
	MSNet (proposed)	75.69	64.93	85.63	97.50	90.94	84.75	91.75	93.63	85.94	85.64	9.99	0.7128	0.039*
Hybrid	Conformer 2023^40^	82.50	65.71	63.75	**98.44**	86.56	**90.31**	87.81	94.38	**92.19**	84.63	11.49	0.6926	0.359
	ADFCNN 2024^43^	79.37	**72.50**	82.81	96.25	**99.37**	84.68	93.43	**95.31**	86.56	87.81	8.40	**–**	0.82
	MSCFormer (proposed)	78.06	71.21	82.75	97.69	96.81	87.81	**94.00**	94.75	88.88	**88.00**	9.10	**0.7599**	**–**

The bold values indicate the best results. The method marked with plus sign (+) are reimplemented.

**Fig. 9:**
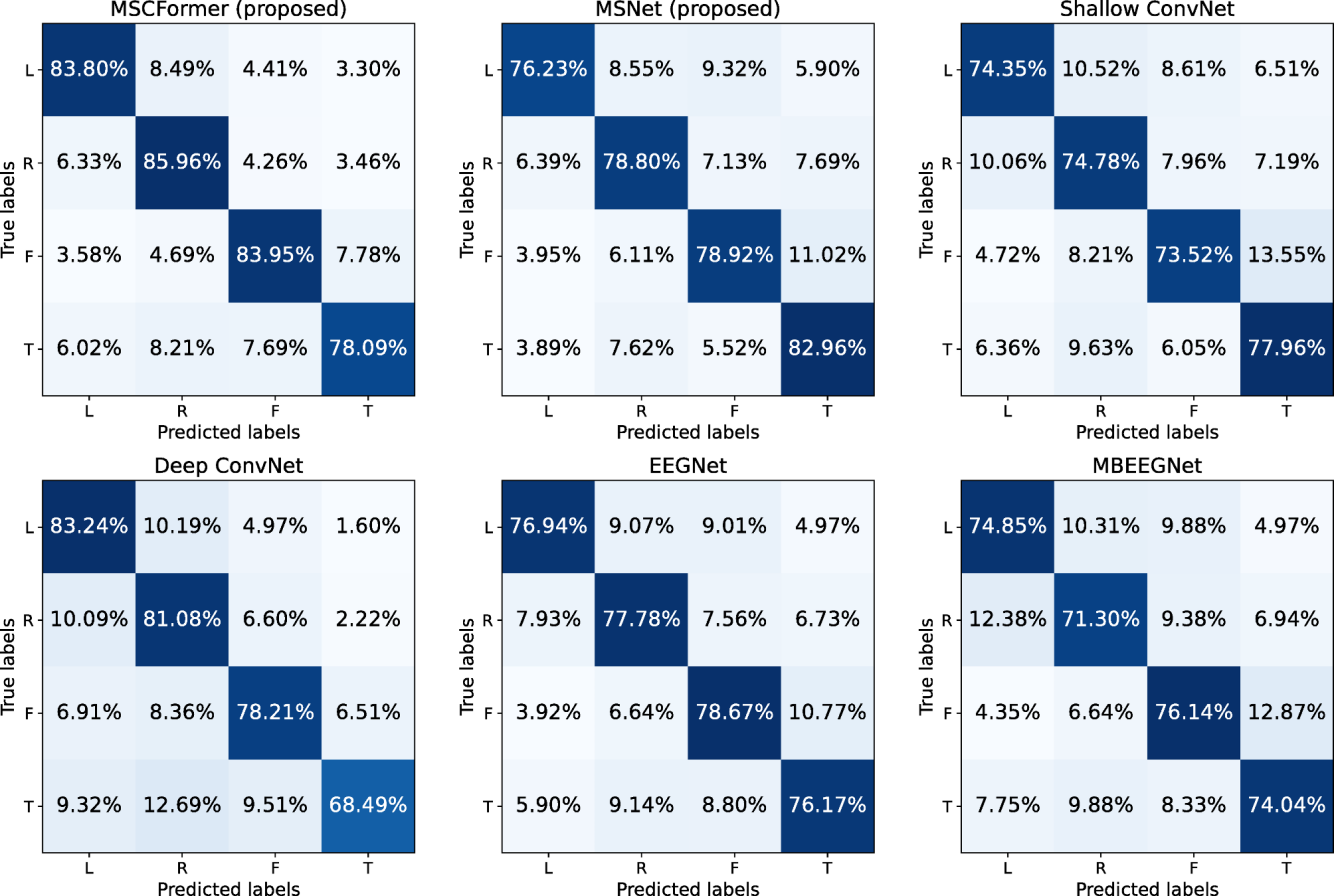
Average confusion matrices of the proposed MSCFormer, MSNet and the reimplemented Shallow ConvNet, Deep ConvNet, EEGNet, and MBEEGNet models. The labels L, R, F, and T in the figure represent the left hand, right hand, feet, and tongue, respectively.

**Fig. 10:**
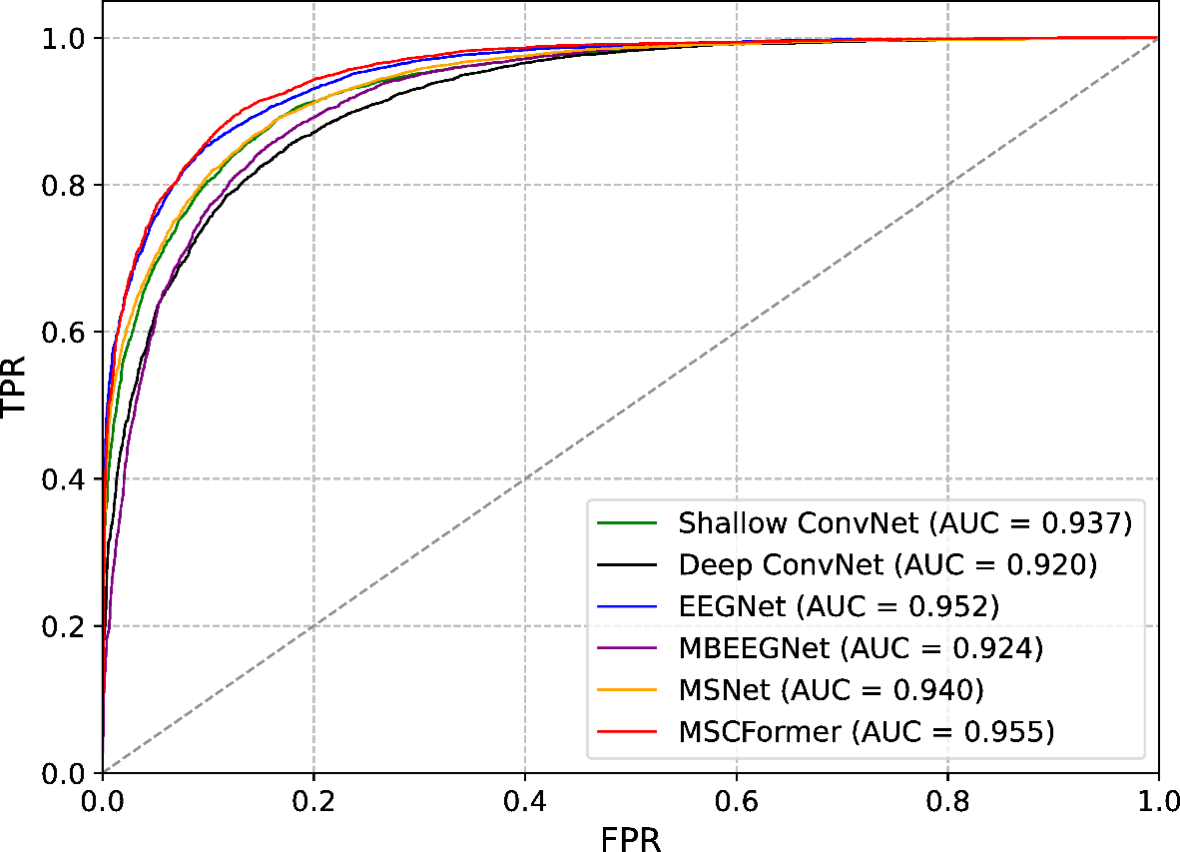
ROC curves and corresponding AUC values for the reimplemented Shallow ConvNet, Deep ConvNet, EEGNet, MBEEGNet, as well as our MSNet and MSCFormer models.

Figure 9 presents the average confusion matrices from five-fold CV across nine subjects on the BCI IV-2a dataset. MSCFormer excelled in decoding the imagined left-hand, right-hand, and feet tasks, achieving accuracies of 83.80%, 85.96%, and 83.95%, respectively. In contrast, the results demonstrate that MSNet achieved the highest accuracy in decoding the imagined tongue task, reaching 82.96%, which is at least 4.8% higher than any other model. These results suggest that while MSCFormer demonstrates overall superiority in most tasks, MSNet may be more effective for certain specific tasks. Figure 10 illustrates the receiver operating characteristic (ROC) curves for these comparison models on the BCI IV-2b dataset, plotted based on their true positive rate (TPR) and false positive rate (FPR) data. Notably, MSCFormer achieves the highest area under the curve (AUC) value of 0.955, surpassing other models.

### Visualization of feature distribution

**Fig. 11:**
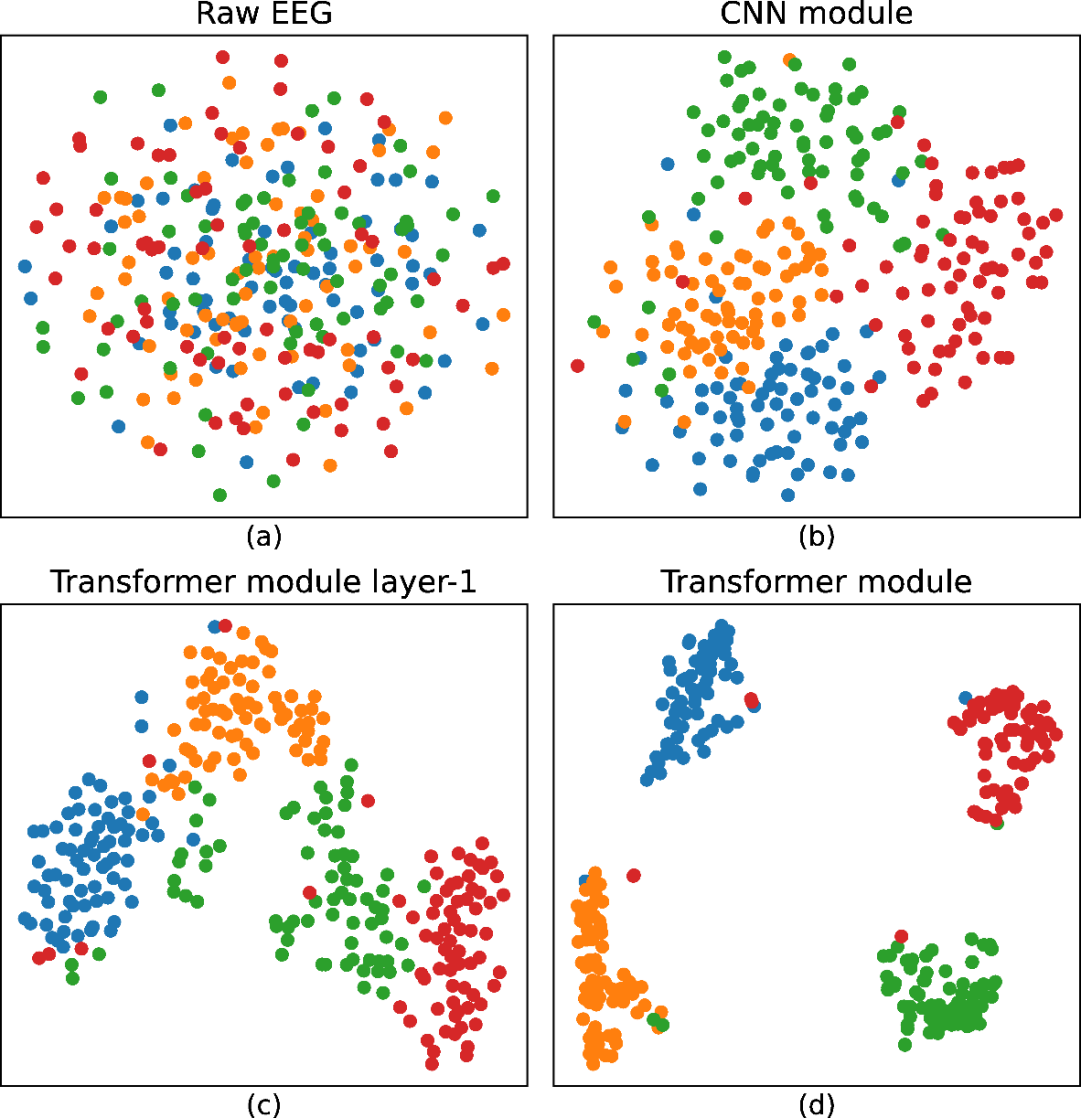
Visualization using t-SNE. (**a**) Raw EEG data distribution. (**b**) Feature distribution after the CNN module. (**c**) Feature distribution after the first layer of the Transformer module. (**d**) Feature distribution following full Transformer integration. Blue dots indicate the left hand, orange dots represent the right hand, green dots represent the feet, and red dots signify the tongue.

To elucidate the discriminatory capacity of the features extracted and enhanced by our MSCFormer model, we employed t-distributed stochastic neighbor embedding (t-SNE) for visualization. This method transforms the high-dimensional features from EEG sequences into a two-dimensional embedding. We visualized the raw EEG data and the transformation at three critical stages of the MSCFormer model: features learned by the CNN module, features enhanced by the first layer of the Transformer module, and features fully enhanced by the complete Transformer module, as illustrated in Fig. 11. The visualization data were derived from five-fold CV models for subject A03. Figure 11(a) presents the raw EEG signal features, where the four class labels are intermingled, making distinctions challenging. Figure 11(b) illustrates that after processing through the MSCFormer’s CNN module, the four categories become discernible, although inter-class boundaries remain blurred, and intra-class distances are still substantial. Figure 11(c) depicts the features enhanced after the first layer of the Transformer module, where inter-class boundaries are more defined, and intra-class distances are notably reduced, underscoring the efficacy of the MHA mechanism in global dependency modeling. Figure 11(d) illustrates the features after full enhancement by the complete Transformer module, where class labels are distinctly segregated, inter-class distances are further enlarged, and intra-class distances are significantly decreased. This demonstrates that increasing Transformer depth significantly enhances MSCFormer’s expressive capability. However, some misclassifications persist, indicating the need for further model optimization to improve accuracy and achieve clearer label separation.

## Discussion

In this section, we will conduct a more in-depth discussion of the ablation study, the impact of hyperparameters on model performance, and comparisons with SOTA methods. Finally, we will discuss the model’s limitations and propose potential areas for future enhancement.

### Discussion on ablation study

This study systematically investigates the impact of the Transformer module, data augmentation, and temporal convolutional kernel sizes in MSCFormer through a series of ablation experiments, where each component is either removed or modified to assess its contribution to model performance. Removing the Transformer module led to varying degrees of performance degradation. Without data augmentation, accuracy on the BCI IV-2a dataset dropped significantly by 5.69% (*p* < 0.01). With augmentation, the BCI IV-2b dataset showed a notable accuracy decrease of 2.36% (*p* < 0.05). This demonstrates the crucial role of the Transformer’s global receptive field in capturing global dependencies and complex patterns in MI-EEG signals, enhancing decoding performance regardless of data augmentation.

Removing data augmentation alone resulted in a 9.57% (*p* < 0.01) and 4.12% (*p* < 0.01) drop in accuracy on the BCI IV-2a and IV-2b datasets, respectively, indicating the importance of data augmentation in increasing the model’s adaptability and coverage across feature space. This effect is especially pronounced for the BCI IV-2a dataset, suggesting that data augmentation significantly enhances model performance in more complex classification tasks.

Removing both the Transformer and data augmentation had the most substantial negative impact, reducing average accuracy by 15.26% (*p* < 0.01) and 5.18% (*p* < 0.01) on the BCI IV-2a and IV-2b datasets, respectively. This substantial drop underscores the combined importance of the Transformer’s global dependency modeling and the diversity introduced by data augmentation.

The selection of kernel sizes (85, 65, 45) in our multi-branch CNN module was inspired by^34^, aiming to capture diverse temporal features in MI-EEG signals. While previous studies, such as^22] and [36^, have used smaller kernels (64, 32, 16), we conducted an ablation study to assess their impact. The results show that replacing our original kernel sizes with smaller ones led to a significant drop in classification accuracy, with decreases of 2.21% (*p* < 0.05) on BCI IV-2a and 1.45% (*p* < 0.01) on BCI IV-2b. This suggests that larger temporal kernels contribute to more effective feature extraction, enhancing MI-EEG decoding performance.

The impact of removing components on the kappa coefficient was similar to that on accuracy. These experimental results reveal that the Transformer module performs global modeling to the multi-scale features extracted from different temporal scales, enhancing the model’s representational capacity and improving decoding performance, while data augmentation ensures robust training in scenarios with limited data.

### Discussion on hyperparameter impact analysis

To investigate the impact of hyperparameters on the performance of the MSCFormer model, we analyzed three key hyperparameters under data augmentation conditions: the depth of the Transformer module, the number of heads in the MHA mechanism, and the pooling size of the CNN module.

The depth of the Transformer encoder plays a crucial role in the model’s ability to capture complex temporal dependencies. Increasing the depth up to a certain point (depth 5) led to an improvement in accuracy, particularly for the BCI IV-2a dataset, where accuracy increased by 2.94% (*p* < 0.05) compared to depth 1. However, further increases in depth introduce the risk of overfitting.

The number of heads in the MHA mechanism also proved to be a critical factor. The eight-head configuration yielded the best performance on both datasets, with notable improvements of 2.58% (*p* < 0.05) on BCI IV-2a and 0.96% (*p* < 0.05) on BCI IV-2b, compared to the single-head configuration. However, adding too many heads (e.g., 24 heads) resulted in diminishing returns, suggesting that excessive splitting of attention may fragment the feature space.

The impact of pooling size showed that an intermediate value optimally balances the retention of temporal features and noise reduction. Pooling sizes of 28 and 44 achieved peak performance on the BCI IV-2a dataset, while a pooling size of 52 was optimal for BCI IV-2b. These results indicate that pooling size should be carefully tuned based on the characteristics of the dataset to avoid information loss or over-smoothing.

### Discussion on comparative with SOTA methods

To comprehensively compare the performance of our proposed MSCFormer with SOTA methods, we analyzed key aspects such as data preprocessing, data augmentation techniques, model architecture, and parameter count, as summarized in Table 5.

**Table 5:** Comparative analysis of our proposed methods and SOTA approaches.

Methods	Preprocessing	Augmentation	Architecture	Parameters		Accuracy %	
				2a	2b	2a	2b
Shallow ConvNet 2017^20^+	STD	S & R	SSCNN	46.1 k	10.8 k	75.15	85.30
Deep ConvNet 2017^20^+	STD	S & R	SSCNN	283.3 k	268.6 k	77.75	83.28
EEGNet 2018^24^+	STD	S & R	SSCNN	**2.9** k	**2.1** k	77.39	87.41
MMCNN 2021^35^	STD	SW & GN	MSCNN + SE	90.3 k	90.3 k	81.43	84.40
MBEEGNet 2022^36^+	STD	S & R	MSCNN	7.1 k	4.7 k	78.69	84.48
MSNet (proposed)	STD	S & R	MSCNN	8.6 k	5.3 k	79.23	86.18
Conformer 2023^40^	BPF & STD	S & R	SSCNN + Transformer	789.6 k	759.2 k	78.66	84.63
ADFCNN 2024^43^	BPF & EEMS	-	MSCNN + Transformer	5.4 k	3.0 k	79.39	87.81
MSCFormer (proposed)	STD	S & R	MSCNN + Transformer	145.9 k	144.9 k	**82.95**	**88.00**

The bold values indicate the best results. The method marked with plus sign (+) are reimplemented.

Across these models, standardization was the most common preprocessing method. The Conformer model applied band-pass filtering before standardization, while ADFCNN incorporated both BPF and electrode-wise exponential moving standardization (EEMS). Most models, except ADFCNN, employed the S&R data augmentation technique, while MMCNN used a combination of sliding window (SW) and Gaussian noise (GN). A notable difference was in the selection of electrode channels: on the BCI IV-2a dataset, MMCNN utilized data from only three channels (C3, Cz, and C4), while other methods utilized data from all 22 channels. Roy et al.^37^ previously investigated the impact of various data augmentation techniques on the BCI IV-2b dataset and demonstrated that the S&R method significantly outperformed GN, SW, and window warping. Additionally, combining these augmentation techniques further improved recognition accuracy. If all models had adopted the hybrid augmentation techniques proposed by Roy et al., their overall performance could have been further enhanced.

The results in Table 5 show that MSCFormer achieved the highest average accuracy across both datasets, demonstrating strong performance in MI-EEG decoding. However, the margin of superiority may have been narrower, or perhaps not the highest, if all models had used the same data preprocessing and augmentation techniques as MSCFormer.

Additionally, although MSCFormer outperforms SOTA models, its high performance comes at the cost of a significantly larger parameter count. This increased complexity enables MSCFormer to capture intricate patterns and long-range dependencies in the data, likely contributing to its superior accuracy. However, the larger parameter count also leads to longer training times and higher computational demands, which may present challenges in resource-limited environments. In practical applications, it is essential to balance MSCFormer’s parameter count with the available computational resources and the potential risk of overfitting. While its complexity improves accuracy, this may not always be practical in scenarios requiring real-time processing or environments with limited computational capacity. Future research could focus on optimizing the model architecture to maintain high accuracy while reducing computational demands, making MSCFormer more adaptable to real-world BCI applications.

### Limitations and future work

While the MSCFormer model proposed in our study outperforms several SOTA methods in subject-specific classification tasks in terms of average recognition accuracy and kappa scores, there are still areas for improvement. First, the MSCFormer model contains numerous hyperparameters, and optimizing them is time-consuming. Key hyperparameters, such as the number of branches in the CNN module and the size and number of temporal convolution kernels within these branches, have not been fully optimized. As a result, the current experimental results may not yet reflect the model’s optimal performance. Future work will focus on automating hyperparameter optimization, integrating it with neural architecture search (NAS) to enable the model to autonomously identify the most effective parameter settings during training, thereby further improving performance. Second, the large number of parameters in the MSCFormer model may limit its deployment on devices with constrained hardware resources. Future research will explore strategies such as model pruning, quantization, and knowledge distillation to reduce the model’s size and computational demands while preserving its high performance. Third, this study primarily addresses subject-specific classification of MI-EEG signals, relying solely on EEG data from individual subjects throughout the training, validation, and testing phases, and excluding cross-subject scenarios. This approach limits the assessment of the model’s generalization capabilities. To address this, future work will focus on applying MSCFormer to cross-subject classification tasks, which will help to evaluate its effectiveness in more diverse application contexts. These efforts could lay a stronger foundation for advancing MI-EEG decoding technology and its practical applications.

## Conclusions

In this study, we introduced MSCFormer, a novel model that integrates a multi-scale convolution module with a Transformer encoder for MI-EEG decoding. The experimental results demonstrated that the multi-branch CNN architecture effectively addresses individual variability in EEG signals by capturing features at different scales. Additionally, the Transformer encoder models global dependencies across these multi-scale features, significantly improving feature representation and classification performance. MSCFormer achieved an average accuracy of 82.95% with a kappa of 0.7726 on the BCI IV-2a dataset, and an average accuracy of 88.00% with a kappa of 0.7599 on the BCI IV-2b dataset, outperforming several SOTA methods. These findings highlight MSCFormer’s ability to enhance MI-EEG decoding performance, establishing a strong foundation for further research into multi-scale feature extraction and global dependency modeling in EEG-based BCI systems.

## Data Availability

The BCI IV-2a and IV-2b datasets analyzed during the current study are available in the BCI Competition IV repository [https://www.bbci.de/competition/iv/#datasets].
